# Contemporary Hermits: A Developmental Psychopathology Account of Extreme Social Withdrawal (Hikikomori) in Young People

**DOI:** 10.1007/s10567-023-00425-8

**Published:** 2023-01-18

**Authors:** Peter Muris, Thomas H. Ollendick

**Affiliations:** 1 Department of Clinical Psychological Science, Faculty of Psychology and Neuroscience, Maastricht University, P.O. Box 616, 6200 MD Maastricht, The Netherlands; 2 Department of Psychology, Child Study Center, Virginia Polytechnic Institute and State University, Blacksburg, VA USA

**Keywords:** Extreme social withdrawal, Hikikomori, Young people, Psychopathology, Developmental transition, Negative family influences, Societal influences, Excessive internet use, Loneliness

## Abstract

Although it is widely accepted that human beings have an ingrained ‘need to belong,’ there seem to be a substantial subset of young people who seclude themselves for most of the time at home and no longer engage in education or work, ultimately withdrawing from participation in society. In Japan, this phenomenon has been labeled as ‘hikikomori,’ but given its global presence it may be preferable to use the term ‘extreme social withdrawal’ (ESW). In this qualitative review, we provide a description and definition of ESW, provide figures on its prevalence, and discuss a number of associated concepts, including loneliness and “aloneliness,” school absenteeism and dropout, the ‘new’ developmental stage of adultolescence, and the labor force categories of freeter (‘freelance arbeiter’) and NEET (a young person not in employment, education, or training). The core of the paper is focused on the origins of ESW in young people and provides a narrative overview of relevant etiological factors, such as aberrant brain processes, unfavorable temperament, psychiatric conditions, adverse family processes including detrimental parenting, negative peer experiences, societal pressures, and excessive internet and digital media use, which are all placed within a comprehensive developmental psychopathology framework. We will close with a discussion of possible interventions for young people with ESW and formulate a guideline that describes (the temporal order of) various components that need to be included in such a treatment.

## Introduction

### Three Cases

Yoshi (30 years) is a Japanese man who for the past 10 years has spent most of his time restricted to his room in the house of his parents. Being their only child, the parents—who successfully run a real estate agency—pampered him a lot during upbringing, catering to his many affectionate and materialistic needs. He has always been shy and usually avoided being in the center of attention. In primary school, he had a couple of friends, but after entering high school these social contacts vanished and he had difficulties connecting with his peers. He was an average student who completed a vocational training program acquiring a certificate as a computer technician. Although he finds it interesting to work with computers, he feels disappointed about the fact that he did not achieve the academic level of his parents who both graduated with a master degree at a business university. His social wariness hindered him during job interviews and after a couple of failed attempts, he gave up and no longer made the effort to apply for work. During that time, he began to withdraw even more and nowadays Yoshi barely leaves his room where even his meals are served by his mother. He devotes most of his time to surfing on the internet. He spends quite a lot of money on online shopping and all of these costs are reluctantly covered by his parents. They continue to hope that at some point Yoshi will apply for a job so that he will earn his own income and make an independent living. Some years ago, Yoshi was seen by a psychiatrist who classified him with social anxiety disorder and a suspicion for autism spectrum problems (based on: Kato et al., 2016).

Aaron (22 years) is a shy young man who is living with his mother and sister in a middle-sized Dutch city. His father died when he was 12 years old following a traffic accident, a dreadful event but one that he reportedly dealt with quite successfully. When Aaron was 18 years old, he was seen by a school psychologist because he experienced some academic difficulties. In high school, Aaron was enrolled in pre-university classes; although he had never been a brilliant student he was always performing on an average level. However, during the final year, the workload increased and his grades started to decline, which eventually resulted in him failing the exams. He regularly skipped classes, complaining about fatigue and not feeling well. The Wechsler test revealed that there was nothing wrong with his intelligence: he had a balanced cognitive profile with a total IQ of 120. He neither appeared anxious to take tests nor reported any attentional problems, but frankly admitted that he was just lazy, profoundly disliking schoolwork, and insufficiently motivated. Instead, he preferred to play computer games, with a lot of practicing during the day and engaging in extensive gaming sessions with some classmates for a large part of the night. Some counseling sessions with the school psychologist were helpful to normalize his daily routine and to prompt him in doing somewhat more schoolwork, which eventually resulted in him passing the exam. After he took a gap year, during which he predominantly hung out with friends and mostly played computer games, he started to study economics in his home city. After half a year, he quit the studies and gradually went back to his old gaming routine with a lot of nightly sessions, this time against unknown international opponents. During the past 2 years, Aaron has been gradually spending more time in his room. Occasionally, he leaves the house to meet his old friends, but this happens less and less as they are increasingly busy with their own life (this case was seen by the first author in his clinical practice).

Chris is the fifth-born child of six children raised by both of his parents, hardworking people living in a rural town in Maine, a state in the northeastern part of the United States. The family was living a quiet life, a bit withdrawn from the rest of the community. Most members of the family were introverted, and this was certainly the case for Chris as well who was described by others as “quiet,” “shy,” and even as “a bit nerdy” because he made a socially inept impression and spent a lot of time on reading. In high school, his grades were good but socially he was quite isolated. This was not because his peers disliked him, but more due to his preference to do things on his own and never join any social events, sports, or clubs. He had two or three friends with whom he liked to hang out in the evenings, but these were rather rare occasions. After graduation, he decided to enroll in a basic electronic course: he was fascinated by computers and the curriculum included computer repair. After finishing this course, he found a job at a company specializing in the installation of alarm systems in houses and cars. He was still living at home and from the money he had been saving from his job he bought himself a used car. However, he was not very happy with his life. His job was not very challenging from an intellectual standpoint and also required him to interact and communicate a lot with other people, something that he did not like at all. He was longing for a more solitary life and thought more and more about escaping from his current life. When Chris was 20 years old, he took the radical decision to act on these thoughts: he drove into a remote area, abandoned his car, and disappeared into the woods, where he lived a secluded existence for 27 years. He survived his chosen exile by committing over 1000 burglaries during which he stole food and other necessary goods from houses in the nearest inhabited world (based on: Finkel, 2017).

All three cases are examples of young people who display some extreme form of social withdrawal. The case of Yoshi was described as a classic example of hikikomori, which was originally seen as a Japanese culture-bound syndrome referring to adolescents and young adults who seclude themselves for most of the time in their parents’ home and no longer are engaged in education or work, thereby refraining from participation in society (Teo, 2010; Teo & Gaw, 2010). Hikikomori is a portmanteau word combining the Japanese verbs of ‘hiku’ (‘to pull back’) and ‘komoru’ (‘to seclude oneself’) that is nowadays used to refer to the syndrome of extreme social withdrawal as well as to the people who display this type of aberrant behavior. As illustrated by the cases of Aaron and Chris—who came from Western countries such as the Netherlands and the USA, it has become clear that the prototypical features of the syndrome are not exclusive to young people from Japan but have been documented in many countries all around the world (Bowker et al., 2019; Teo et al., 2015a). Illustrative in this regard is the international investigation by Kato et al. (2012) who presented case vignettes of hikikomori to 247 psychiatrists and psychiatric trainees. About half of the respondents were from Japan, whereas the others came from other (Western and Eastern) countries. Their main instruction was to indicate whether they had ever encountered patients with a similar clinical profile in their daily practice. Respondents indicated that patients with a hikikomori profile are encountered in all countries, with no differences in estimated prevalence rates between Japan and the other countries. Altogether, these findings signify that hikikomori is not solely a Japanese, culture-specific syndrome but can be regarded as a global phenomenon (Li & Wong, 2015a; Wu et al., 2019). This is also why—in the further course of this review—we prefer to use the term ‘extreme social withdrawal’ (ESW) over the Japanese label of hikikomori, although we occasionally refer to the latter label when discussing relevant literature. Other terms that can be used are ‘severe social withdrawal’ or ‘prolonged social withdrawal’; these also reflect the seriousness of the problem as well as the global presence of the phenomenon and hence can be considered as appropriate alternatives to ESW.

The present review mainly discusses ESW from a clinical perspective, which implies that this type of behavior is considered as negative and ‘aberrant’ and hence needs to be corrected in some way. However, it is good to note that there may be also positive motives for seeking solitude (Finkel, 2017). For instance, in the past, people who sought seclusion oftentimes had a religious motive. These individuals were commonly named ‘hermits’ who isolated themselves from the community with the aim to contemplate and pray, which would bring them in closer contact with God. Such an ascetic lifestyle was generally seen as something admirable and worthy of pursuing and a number of these hermits were later canonized to become saints. As another example, there are also persons who are deeply disappointed by society. They are idealists who prefer a solitary existence because they can no longer bear to live with others who willingly comply with the current societal norms and values, which include egocentrism, consumerism, and materialism (see Boyd et al., 2012).

Despite the fact that ESW can have positive motives, all three cases described above show that it is difficult for ‘contemporary hermits’ to survive in present society. Yoshi and Aaron were fully dependent on the support and financial resources of their families and so it will be difficult for them to continue with their secluded life once this sustenance ceases. An extreme example of this is the ‘8050 crisis’ in Japan, which refers to the phenomenon that parents entering their 80s, because of physical, cognitive, and financial restrictions, can no longer take care of their hikikomori offspring (who have been living in seclusion for many years and are already in their 50s; Yamazaki et al., 2021). Chris did not rely on support of others; he survived his self-chosen exile by committing multiple crimes, as a result of which he was ultimately prosecuted in the legal justice system (see also Chan, 2019). Given associated difficulties, we tend to consider ESW as a clinically relevant problem. Previous reviews have appeared on the topic providing excellent overviews of the phenomenon that addressed various factors that play a role in its etiology (e.g., Kato et al., 2019; Li & Wong, 2015b; Nonaka et al., 2022; Teo, 2010). However, none of these have explicitly taken a developmental psychopathology perspective (Cicchetti, 2006) which, we assert, provides an optimal framework for understanding the origins and course of this aberrant behavior.

This qualitative review summarizes the current knowledge on ESW, although it should be acknowledged that it is mainly based on the English-language literature and as such excludes a significant amount of Japanese studies on this topic. Fortunately, Japanese scholars have also published in English, so that we still have a good impression of their scientific work on this phenomenon. The review starts with a description of the normative progression of social development in young people. Then, we provide a definition of ESW and present some epidemiological figures. Next, we will address a number of psychological constructs that are closely related to ESW. Then we will provide a detailed narrative of the factors that are involved in the etiology and maintenance of ESW and place these within a comprehensive developmental psychopathology framework. Finally, we will close by delineating treatment components that—in our view—should be included in interventions for young people with ESW.

## Normative Social Development

The phenomenon of ESW is difficult to reconcile with what has been labeled by Baumeister and Leary (1995) as ‘the need to belong’, which is considered to be a fundamental motivation of human beings and can briefly be defined as a basic desire to form and maintain enduring interpersonal attachments and relationships. The need to belong already shows itself at a very young age by innate, reflex-like responses such as crying and smiling which ensure the proximity of the parents and hence serve the purpose of survival (Schaffer, 2003). Infants will subsequently develop more sophisticated communicative behaviors including gaze following, reciprocal verbal exchanges, and shared attention, to eventually form an attachment bonding with their primary caregivers. It is assumed that, during the further course of development, this secure attachment relationship will enable youngsters to forge lasting social connections with others (Bowlby, 1969/1982), thereby satisfying their desire for belongingness. Normally, the parents constitute the primary social connection in younger children, but as they grow older the school including teachers and fellow students are an important sphere of belonging. In adolescence, the peer group (at school and in the community) and the formation of friendships become more dominant and with the transition to adult life the social connections at work and romantic partners are increasingly important as sources to gain a sense of belongingness (Allen & Kern, 2019).

According to Erikson’s (1959) psychosocial theory, adolescence is a crucial period in the development of identity and the transition toward adulthood. More specifically, between the ages of 12 and 18 years, youth frequently engage in personal and interpersonal experimentation to find out who they are and what to do in life. Personal beliefs, goals, and values are explored, adjusted, and eventually anchored, leading to an establishment of one’s own identity. When youngsters succeed in this, they will achieve the virtue of “fidelity,” which is characterized by a strong sense of self-esteem, an ability to operate independently from others in accordance with their individuality, but also a flexible adjustment to the norms, values, and expectations of the external world. However, when young persons’ fail to achieve this status, they will find themselves in a state of role confusion, which means that they experience extreme doubts regarding their own identity as well as the meaning and purpose of their existence, leading to isolation and a sense of being lost (Erikson, 1968).

From a biological point of view, it is important to recognize that the human brain can be distinguished from the brain of other species largely because of its social potential: human beings are capable of ‘reading’ others’ states of mind, evaluating others on the basis of emotions, and using all this information to flexibly respond to and interact with other people (Adolphs, 2009). For this purpose, the human brain is geared with a complex and advanced network that makes it possible to evaluate the intentions, desires and beliefs, feelings, dispositions, and actions of other people. This network, which is also known as the ‘social brain,’ includes brain areas, such as the amygdala, medial prefrontal cortex, anterior cingulate cortex, inferior frontal gyrus, and the anterior insula. It is generally assumed that many parts of the social brain undergo functional and structural development during adolescence, which lead to a substantial improvement of social-cognitive abilities (Blakemore, 2008). In a similar vein, Crone and Dahl (2012) also consider brain development as a crucial element in the normative social development of young people. These scholars describe adolescence as a developmental stage during which, due to rapid surges in hormone levels at the onset of puberty and subsequent changes in the limbic circuits (including the amygdala and the ventral striatum), social-cognitive engagement increases (e.g., the young person begins to attach greater importance to receiving attention and admiration from and being accepted by peers; see also Blakemore & Mills, 2014). The maturation of the frontal brain areas related to the social brain network and those involved in the exertion of cognitive control increasingly enable the adolescent to engage in socially competent and goal-directed behavior. However, as a result of aberrant brain processes and/or negative social learning experiences (e.g., ostracism, bullying), the social development may also take a negative growth trajectory that is characterized by social withdrawal, loneliness, and depression (see Crone & Dahl, 2012).

Taken together, normally, young people are intrinsically motivated to connect to others, gradually form an identity that provides them with a solid basis for participating in adult social life, and their brain develops in such way that it is optimally geared to process social information. These perspectives of normative social development also provide ways for looking at young people who display ESW. More specifically, these youngsters may lack the fundamental motivation for initiating and maintaining enduring social relationships, are somehow stuck in their identity development and as such fail to make the psychosocial transition to adulthood, and/or have difficulties to effectively deal with the biological and environmental social-affective challenges posed by adolescence, all of which hinder them to develop socially and may prompt them to disengage from social life.

## Definition of ESW

Social withdrawal refers to the process whereby children and adolescents remove themselves from social life and no longer engage in interactions with other people (Rubin et al., 2009). Obviously, social withdrawal reflects a continuum with ESW on its extreme end.

In the hikikomori literature, the prolonged avoidance of participation in social life is considered as the key feature of ESW: the person is spending time most of the day and nearly every day at home, mostly seeking the privacy of the bedroom. The person is no longer participating in social routine activities such as attending school and going to work and maintains only minimal interpersonal relationships. The Japanese Ministry of Health, Labor, and Welfare (2003) was first to formulate clear criteria for the condition. These included “(1) A lifestyle centered at home; (2) No interest or willingness to attend school or work; (3) Persistence of symptoms beyond six months; (4) Schizophrenia, mental retardation, or other mental disorders have been excluded; and (5) Among those with no interest or willingness to attend school or work, those who maintain personal relationships (e.g., friendships) have been excluded” (see Teo, 2010; p. 180).

In the past decades, the criteria of ESW have evolved and although there is still no general consensus, the most common definition now contains the following three key elements (Kato et al., 2019): (1) Marked social isolation in one’s home, which includes the physical withdrawal in one’s own place of residence, the absence of active participation in academic and labor settings, as well as the limited involvement in social relationships, (2) The duration of the social isolation is at least six months, and (3) The social isolation is associated with significant functional impairment or distress.

With regard to the first element, the suggestion has been made to indicate the severity of the social isolation: persons occasionally leaving the home to interact with others (2–3 days per week) can be qualified as ‘mild,’ those who rarely leave the home and barely interact with others outside (1 day per week or less) can be classified as ‘moderate,’ whereas individuals who remain in their room and minimally interact even with cohabiting family members can be defined as ‘severe’ cases (Kato et al., 2020a). Relatively mild cases of ESW have also been labeled as ‘quasi-hikikomori’ (Tajan et al., 2017), ‘semi-hikikomori’ (Su et al., 2021), or ‘soft subtype hikikomori’ (Pozza et al., 2019): these terms are used for individuals who spend most of the time alone in their home, yet occasionally go out to conduct a hobby or hang out with others.

The second element (i.e., the duration of at least six months) is not uncommon in the definition of mental health problems (American Psychiatric Association [APA]). Note, however, that the choice of this time period is rather arbitrary. Tajan et al.’s (2017) analysis of the 2016 epidemiological survey on Japanese hikikomori revealed that in the majority of cases (i.e., 75%), the withdrawal lasts for more than 1 year and that almost half of these individuals even report a time span of more than 7 years. This indicates that ESW should be seen as a rather persistent and refractory problem.

The third and final element of functional impairment/distress was added to the definition of ESW to reflect the problematic nature of the phenomenon, underlining that persons displaying this behavior are in need of professional help and intervention. This is nicely illustrated in a study by Teo et al. (2015a) who examined the psychosocial functioning of 36 adolescents and adults with hikikomori. The results showed that these secluded individuals displayed high levels of loneliness, had weakly developed social networks, and showed considerable levels of disability. Moreover, there is evidence indicating that individuals displaying ESW are at increased risk for committing suicide (Yong & Nomura, 2019; Zhu et al., 2021). All this suggests that at least on a group level, ESW is associated with substantial psychosocial impairment and disability. However, it is good to be aware that there may also be individuals who—despite objective dysfunctioning—are perfectly happy with their chosen isolation and that “being ‘hidden’ is [their] preferred lifestyle” (Chan & Lo, 2014b; p. 951).

## Prevalence

So far, most information on the prevalence rates of ESW has been gathered in Japan. In the World Mental Health Japan Survey that was conducted between 2002 and 2006 (Koyama et al., 2010), 4134 respondents from various urban and rural areas in this Eastern country were included. A subsample of respondents with an age between 20 and 49 years (*n* = 1660) were asked whether they had ever been “Staying in the house continuously for more than 6 months, not going out to work or school and having little communication with people other than your family.” It was found that 19 respondents endorsed this question positively and hence indicated to have experienced hikikomori during their life, which suggests a lifetime prevalence of approximately 1.2%. The prevalence in males was more than four times larger than in females (i.e., 1.8% versus 0.4%). The vast majority of the respondents (i.e., 82.5%) reported that the period of seclusion had begun during their adolescence (10–19 years: 57.5%) or young adulthood (20–29 years: 25.0%), which justifies the present review’s focus on young people with ESW. However, Koyama et al. noted that there also seems to be a substantial minority (13.8%) with an age of first onset in their midlife years (40–49 years), during which people typically begin to reflect on their life which can possibly put new strains on their mental health. In a second wave of the World Mental Health Japan Survey, which was conducted between 2013 and 2015 with 1776 respondents aged 20 to 64 years, 2.2% was defined as suffering from lifetime hikikomori (Nishi et al., 2019), which led the authors to conclude that “the prevalence of hikikomori has increased in the past 10 years” (p. 460). Prevalence rates of ESW in other countries are sparse (Wong et al., 2015) but there are clear indications that this phenomenon is common in countries in the Western part of the world as well (Kato et al., 2012).

## Related Concepts

### Withdrawal Prone Temperament or Personality

There are clear individual differences in the extent to which people are desirous of and willing to engage in activities with other people. Most personality theories cover this with the trait of extraversion-introversion, where extraversion refers to the tendency to be active, outgoing, assertive, and talkative and introversion pertains to the inclination to be calm, reserved, shy, and quiet (Eysenck, 1952). Extraversion and introversion are opposite traits that are part of one and the same dimension. Persons who are more extravert are strongly socially oriented: they engage actively with others to acquire friendship, love, admiration, and status. Persons who are more introvert prefer to stay more in the background and occasionally retire from social situations to avoid overstimulation (Burger, 1995; Schmeck & Lockhart, 1983). These individuals typically have a tendency to withdraw and seek social isolation in a quiet place (Oishi & Choi, 2020).

This does not automatically mean that all introvert people are ‘loners’ and display ESW. Most of them do have a social desire and hence establish and maintain social relationships. However, those introverts who are also more easily distressed, anxious, and shy seem to be more susceptible to become trapped in social disengagement and withdrawal (Tuovinen et al., 2020). In the child psychology literature, young people with such a temperament constellation have been described under the label of ‘behavioral inhibition’ (Kagan, 1994; Muris & Ollendick, 2005). Behavioral inhibition has been defined as the tendency to react with fear and avoidance toward unfamiliar stimuli, situations, and people. There is abundant evidence that this early temperament characteristic (which occurs in its extreme in 15% of youth) constitutes a significant vulnerability factor in the development of psychopathological conditions that are associated with severe social dysfunctioning, including social anxiety disorder (Clauss & Blackford, 2012) and selective mutism (Gensthaler et al., 2016), and so there is reason to assume that inordinate introversion and behavioral inhibition in specific might also play a role in ESW.

### Loneliness and “Aloneliness”

Loneliness can be defined as an unpleasant emotional response to the perception that the quantity or the quality of one’s social relationships is insufficient. This perception is subjective: some people live a solitary life but do not feel lonely, whereas others have quite a number of good friends and still feel lonely nonetheless (Hawkley & Cacioppo, 2010). Whereas loneliness is concerned with the perception of too much solitude, aloneliness pertains to the perception of not enough solitude (Coplan et al., 2021). More precisely, aloneliness has been conceptualized as a negative feeling that arises from the perception that one is not spending enough time on his or her own (Coplan et al., 2019a). The contrasting concepts of loneliness and aloneliness are both related to social withdrawal tendencies, but the links are quite complex and seem to be dependent on underlying social motivations.

This point can be illustrated by means of an interesting study by Coplan and colleagues (2013) who explored the relations between social motivation, social withdrawal, and socioemotional functioning in children aged 9 to 12 years. Based on social motivations, four groups of children were identified. Three groups were defined as socially withdrawn: (1) a shy-conflicted group that consisted of children with high shyness scores and a low-to-moderate preference for solitude; (2) an unsociable group that was composed of children with low-to-moderate shyness scores and a strong preference for solitude; and (3) an avoidant group that contained children with high shyness scores and a strong preference for solitude. The fourth ‘non-withdrawn’ control group consisted of children displaying low-to-moderate scores for both shyness and the preference for solitude. When looking at the psychosocial functioning of these groups, it was found that the shy conflicted and in particular the avoidant children experienced elevated levels of psychosocial problems (e.g., negative affect, anxiety, and depression), whereas the unsociable children did not differ from the non-withdrawn control children.

Similar findings have been documented in adolescents and young adults (Barzeva et al., 2019; Coplan et al., 2021) leading to the main conclusion that social withdrawal constitutes a quite heterogeneous umbrella construct. It harbors young people who due to strong avoidance and/or unmet approach tendencies suffer from too little social contact, hence feel lonely and are prone to develop various types of internalizing psychopathology, but also includes individuals who have fewer social needs and want to be alone (Coplan et al., 2019a). It should be noted that many studies on ESW have neglected this heterogeneity, but it is good to keep in mind that for some young people social withdrawal is not a negative experience that is associated with feelings of loneliness. In these cases, the retirement from the social world may be fueled by feelings of aloneliness and thus reflects a willful attempt to seek solitude. The case of Chris presented at the beginning of this article seems to be a good illustration of this.

### School Absenteeism and School Dropout

Prolonged absenteeism from school may constitute an early indicator of ESW. When looking at the school absence continuum, there are young persons who show mild school-avoiding behaviors such as repeated tardiness in the morning or occasionally skipping classes ranging to those who are completely absent from school for an extended period of time (Kearney, 2008) or even completely drop out without acquiring a certificate or diploma (Alexander et al., 2001). Various motives have been put forth that help explain why youth decide to avoid school (Kearney, 2008). These include avoidance of the distress associated with typical school-related activities, such as traveling in the bus, entering the school, and changing classrooms. Other youngsters evade school because of social or evaluative reasons: they experience difficulties when interacting with peers and/or fear performance situations, such as exams, oral presentations, and sports (see also Inoue et al., 2018). Besides these negative motives, there may also be positive incentives that make it more appealing to be at another place than school. For example, some young people like to engage in outdoor activities with a friend or prefer to stay at home to watch television, surf on the internet, or play videogames.

In the USA, the chronic absenteeism rate, which has been defined as missing 10% or more of the regular school days in a year, is close to 15%, whereas the prevalence of dropout is about 5% (Irwin et al., 2022). It is good to keep in mind that not all of these youths meet the criteria for ESW (the figures include, for example, truant adolescents who skip school to hang out with friends or young persons whose parents provide little supervision on school attendance), but at least there appears to be some overlap between these school-related concepts and prolonged social withdrawal.

### NEETs and Freeters

The phenomenon of NEET refers to individuals who—after termination of compulsory schooling—are ‘not in employment, education, or training.’ In an interesting study, Uchida and Norasakkunkit (2015) obtained evidence indicating that there are clear communalities between NEETs and those showing ESW. These researchers developed an instrument, the NEET-Hikikomori Risk (NHR) scale that measures (a) a negative attitude toward school and work, (b) lack of self-competence, and (c) unclear ambition for the future, all of which are features of young people who show a tendency to deviate from the societal mainstream. The newly developed scale was then completed by 7,725 Japanese participants from the general population aged between 20 and 39 years, of whom a small minority were classified as NEETs (0.8%) or persons’ who displayed ESW (1.06%). It was found that participants with ESW exhibited the highest scores on the NHR scale, closely followed by the NEETs, who in turn had higher scores than the others, with all the groups being significantly different from each other. These results support the notion that ESW and a NEET status are both indicators of young persons’ being marginalized by society and hence seem to be part of one and the same continuum (Liew et al., 2021).

A ‘freeter’ (which is a word derived from ‘free timer’ or ‘freelance arbeiter’) can be regarded as a ‘light’ version of a NEET and has been defined as a person who has not started a proper working career after high school or university, but does earn some money from part-time, low-skilled, and low-paid jobs (e.g., working in a restaurant, supermarket, or shop). Being a freeter is not necessarily problematic: some young individuals adopt this lifestyle because they go for a career that is less compatible with the societal standard (‘dream pursuing’ type; e.g., becoming a singer or an actress) or just want to enjoy life for some time before starting the ‘rat race’ of a working career (‘moratorium’ type; Kobayashi, 2011). However, in case the freeter has schooling, dropout, and marginalization issues and hence is forced to take on occasional, simple, low-paid jobs (‘no alternative’ type), he or she may approach to the status of a NEET.

### Adultolescence

Adolescents and young adults who seek isolation in the comfort of their home and do not take part in dutiful activities such as going to school or work are often accused of shifting their responsibilities in life and not taking matters into their own hands, which can be qualified as signs of immaturity (Kato et al., 2016) or even a rejection of conforming to the current cultural standard of becoming a socially sanctioned adult (Uchida & Norasakkundit, 2015). Meanwhile, it should be kept in mind that society has changed considerably during the second half of the twentieth century and that this has had also consequences for the development of young persons and more in specific the timing of adulthood.

For example, Furstenberg et al. (2003) used data from the USA census to retrieve a number of objective milestones that are associated with becoming an adult and which jointly define when young people have accomplished the transition to adulthood. The milestones included leaving home, finishing school, becoming financially independent, getting into a stable romantic relationship, and having a child. These authors compared the percentages of young persons who completed the adult transition in 1960 and 2000 by the ages of 20, 25, and 30. It was noted that in 1960, adulthood was reached by 19% of the young people at the age of 20, 56% at the age of 25, and 71% at the age of 30. In 2000, all these percentages were remarkably lower (i.e., 4%, 19%, and 38.5%, respectively), indicating that the transition to adulthood no longer seems to take place at the end of adolescence but appears to be postponed toward the fourth decade of life. Further studies indicate that this trend is still relevant for current generations of youngsters and not only occurs in the USA but also in other affluent Western and Eastern countries.

On the basis of this observation, developmental psychologists have pleaded for the implementation of a new developmental stage bridging the years between adolescence and adulthood, which has been labeled as ‘quasi-adulthood’ or ‘adultolescence’ (Furstenberg et al., 2003; Waters et al., 2011), which are terms that are sometimes used with a condemnatory flavor as they imply that young people are considered to be somehow ‘stuck’ in their process of growing up, and this would be particularly true for those who display ESW (Saito, 2013).

## Origins of ESW

It may be clear by now that ESW is a complex phenomenon and this is also true for its etiology. It is assumed that young people’s tendency to withdraw and seclude themselves has a multifaceted origin, which means that many potential factors are involved in the emergence and maintenance of the phenomenon. In this section, we provide an overview of the relevant factors beginning with psychiatric disorders that are associated with an inclination to seek prolonged social isolation (see also Kato et al., 2019; Masi et al., in press), after which we proceed to explore a number of environmental influences and societal issues that may be associated with ESW.

### Psychiatric Disorders

Briefly, ESW can occur as a concurrent condition of other mental health problems. In the hikikomori literature this is often referred to as ‘secondary hikikomori’: in these cases, the persistent social withdrawal symptoms clearly occur within the context of another disorder. Some scholars assume that there is also primary or idiopathic hikikomori, which arises without a clear history of psychiatric comorbidity (Teo et al., 2015a). In these cases, a specific constellation of personal, family, and societal characteristics seems to be present that predispose the young person to exhibit ESW (e.g., Suwa & Suzuki, 2013). However, the boundary between primary and secondary types of ESW is not clear (Frankova, 2019) and with the growing acknowledgment of the dimensionality of most forms of psychopathology (APA, 2022), it has become increasingly difficult to rule out the presence and exact contribution of psychiatric conditions to ESW.

Table 1 provides a summary of what is known about the relationship between ESW and the five most plausible psychiatric conditions that are associated with persistent withdrawal tendencies, namely schizophrenia (and other psychotic disorders), social anxiety disorder, depression, autism spectrum disorder, and avoidant personality disorder. It can be concluded that ESW is associated with each of these disorders and for this reason scholars consider social withdrawal as an important transdiagnostic construct (Masi et al., in press; Porcelli et al., 2019). However, its exact role in various types of psychopathology is less clear: in some cases, ESW appears to be (an extreme variant of) a pathognomonic symptom of the disorder (e.g., schizophrenia, ASD), while in other cases ESW constitutes the ultimate consequence of a deranged disorder (e.g., social anxiety disorder, depression), which in turn can act as an antecedent variable accounting for the continuation of the psychiatric problem (Oliva et al., 2022).

**Table 1:** The relation between ESW and various psychiatric conditions

Psychiatric condition and proposed link with ESW	Empirical evidence study	Results
*Schizophrenia or other psychotic disorders*: Besides positive symptoms (e.g., delusions, hallucinations), the disorder is characterized by negative symptoms, which include asociality: the diminished interest in and engagement in social relationships	Kondo et al. (2013): 148 clinically referred adolescents and young adults aged 16–35 years with hikikomori	12.2% fulfilled DSM criteria of schizophrenia or another psychotic disorder
	Chauliac et al. (2017): 66 patients aged 18 to 34 years who were visited at home by psychiatric outreach team	37% of the patients met ICD criteria of a psychotic disorder
	Malagón-Amor et al. (2018): 190 cases with a mean age of 39 years who were subjected to home visitation program because regular outpatient treatment was no longer feasible	With 37.6%, psychotic disorder appeared to be most prevalent psychiatric condition
	Yasuma et al. (2021): Community sample of 1616 adults with an average age of 37 years, some of which showed clear signs of hikikomori	Relationship between hikikomori and psychotic symptoms (in specific delusions)
*Social anxiety disorder (SAD)*: Extreme fear when meeting (new) people and attending social gatherings because they fear being judged or scrutinized by others and as a result marked avoidance of such social situations	Nakamura and Shioji (1997): 24 consecutively treated patients with taijin kyofusho, a Japanese, culture-specific variant of SAD	29% of the patients with taijin kyofusho met the criteria of hikikomori
	Nagata et al. (2013): 141 adult patients with a mean age of 28 years who were diagnosed with SAD and sought treatment in an outpatient clinic	19% of the patients fulfilled the criteria of hikikomori
	Teo et al. (2015b): 22 people from Japan and the USA with a mean age of 29 years who displayed persistent ESW	27% of the participants with a history of ESW was diagnosed with SAD
*Depression and associated conditions*: Social anhedonia – an increased disinterest in social interactions and lack of pleasure in social situations – is a prominent feature	Koyama et al. (2010): 19 people with hikikomori who had been identified in a large Japanese community survey	32% had experienced a mood disorder during their lifetime; hikikomori individuals had a 6 times higher risk of mood disorder
	Kondo et al. (2013): See above	18% were diagnosed with a mood disorder
	Teo (2012): Case study of a 30-year-old man with bipolar disorder	ESW exclusively occurred during the depressive episodes of the disease and not during manic episodes; successful treatment of depression abolished ESW
	Teo et al. (2015b): See above	32% was diagnosed with major depressive disorder, 18% with dysthymic disorder, and 9% with bipolar disorder
	Teo et al. (2020): 67 patients with major depressive disorder of whom some displayed hikikomori and others did not exhibit this extreme form of social withdrawal	Levels of depression were comparable in both groups, but depressed patients with hikikomori had experienced more prior episodes of depression and reported higher levels of suicidal ideation
	Kubo et al. (2022): 800 Japanese office workers aged 30–59 years who were followed during the first year of the Covid-19 pandemic	Symptoms of (modern type) depression prospectively related to symptoms of ESW and vice versa, which suggests reciprocal relation
*Autism spectrum disorder (ASD):* a neurodevelopmental disorder that is characterized by persistent deficits in social communication and social interaction among which social aloofness	Kondo et al. (2013): See above	37% was diagnosed with disorders usually first diagnosed in infancy, childhood, and adolescence, which included pervasive developmental disorder, the DSM-IV classification of ASD
	Katsuki et al. (2020): 103 adult patients with hikikomori and 221 clinical controls	Hikikomori patients scored higher on a self-report measuring autistic traits and rated themselves as more depressed and lonely
	Shimono et al. (2021): 272 Japanese undergraduate and graduate university students with a mean age of 19 years	Positive relations were found between autism-related difficulties in social interaction and the propensity toward social withdrawal
*Avoidant personality disorder*: A pervasive pattern of social inhibition, feelings of inadequacy, and hypersensitivity to negative evaluation, which ultimately leads to restricted interpersonal contact and social isolation	Kondo et al. (2013): See above	22% was diagnosed with a personality disorder and it is plausible to assume that avoidant personality disorder was one of the most prevalent
	Teo et al. (2015b): See above	41% of the participants with a history of ESW was diagnosed with avoidant personality disorder
	Hayakawa et al., (2018, Study 2): 55 individuals with hikikomori (aged 16–49 years) and 78 age-matched healthy controls	Hikikomori participants displayed higher scores on the avoidant personality scores on the Structured Clinical Interview for DSM–Personality Questionnaire

### Societal Issues

Contemporary young people face a number of challenges that are closely tied to the way the current society is organized (Furstenberg, 2010; Settersten et al., 2006). For example, in the economic climate of capitalism and in specific neoliberalism, education is regarded as an important asset for developing and improving business and industry and as a result many young people currently feel pressured to aim for completing study at the highest possible educational level (Bessant et al., 2017). Furthermore, due to globalization, young people experience greater competition when applying for a job, whereas on the other hand employers mainly offer temporary positions to screen their work potential before giving them a permanent position, so that they are able to adjust quickly to changing market conditions (Churchill & Khan, 2021). Because of the extended investment in education and the more uncertain labor market position, young people have limited financial resources. This means that they are less in the position to acquire housing for themselves and hence are forced to live longer at home with their parents (Forrest & Yip, 2013; Sompolska-Rzechula & Kurdys-Kujawska, 2022). In the popular media, this has been labeled as the ‘full nest syndrome,’ a term that makes clear that the phenomenon can be accompanied by stress and tension for those involved (i.e., parents and offspring). A final struggle concerns the adequate handling of digital media, which are indispensable in today’s society. In particular the younger generations spend a considerable amount of time on electronic devices such as notebooks, iPads, and smartphones and it can be challenging for them to find good balance between real-life activities (school, work, sports, social activities, sleep) and the continuing flow of digital information and all the temptations of the virtual world (Dimock, 2018).

The strains posed by the current society may not only slow down young people’s progression into adulthood but also create the basic conditions for some of them to get stuck in their development and—in extreme cases—to gradually distance and withdraw themselves from the world. School absenteeism, school dropout, as well as freeter and NEET status can be first signs of adolescents and young adults who are on an aberrant trajectory. It is important to note that growing up in an individualized, competitive, and complex world should only be seen as a potential prerequisite for such a scenario: these strenuous features of society are especially taxing for young people who already face adverse personal circumstances, such as the presence of a psychiatric disorder (Gariépy et al., 2022; Gubbels et al., 2019) or socioeconomic inequality (Lörinc et al., 2020; Sosu et al., 2021).

Meanwhile, Varnum and Kwon (2016) pointed out that ESW in young people critically depends on the availability of public resources. More precisely, they argued that from an evolutionary perspective, withdrawal from social life and declining from work for a longer period of time is not a strategy that will enhance one’s chances of survival. So, the fact that ESW does exist in a society is proof of wealth and abundant resources. Apparently, a solid social safety net and/or a family with sufficient financial assets are present (see also Nonaka & Sakai, 2021), which makes it possible for young persons to survive in spite of withdrawing from economic and social life. In societies characterized by a scarcity of resources, young people will need to deploy different methods to deal with their marginalized position, such as theft, prostitution, or drug trafficking, rather than displaying ESW as this would result in a quick elimination (Varnum & Kwon, 2016).

### Environmental Influences

#### The Role of Family and Parents

As many individuals with ESW live their life within the shelter of their family, one wonders about the role of parents in the origins of this phenomenon. Putting it in other words, why would parents allow their offspring to refrain from school or work and stay at home most of their time, thereby preventing young people to build their own life? Part of the answer to this question may lie in the fact that a substantial proportion of the parents of young individuals with ESW have psychiatric problems themselves and as a result have less awareness of the problems of their children and/or are less capable of applying effective parenting behaviors. This possibility is illustrated in the study of Malagón-Amor and colleagues (2020) who investigated the family background of 190 adult participants (mean age 39 years) showing persistent social withdrawal. It was noted that 59.7% of the mothers (most commonly an affective or anxiety disorder) and 19.5% of the fathers (most frequently a psychotic or substance use disorder) had a psychiatric history (see also Umeda et al., 2012). Moreover, in 61.5% of the cases, dysfunctional family dynamics were clearly present. These families were characterized by high levels of conflict and instability, poor communication, excessive control, lack of empathy, and excessive criticism, which are all features that potentially endanger the development and emotional well-being of the individual family members (Hattori, 2006). In 20.7% of the cases, indications were even found for a history of maltreatment within the family. Finally, some indications were found that the adverse family-related variables were positively associated with severity indexes of ESW. Thus, the more dysfunctional the family interactions, the earlier the onset of the social isolation, the less cooperation with treatment, and the lower the overall daily functioning.

Another relevant study was conducted by Suwa et al. (2003) who examined the characteristics of Japanese families of adult children with ESW who were still living at home (mean age 30 years). Parents of offspring who showed clear social withdrawal symptoms (*n* = 27) and a control group of parents with offspring displaying no signs of social withdrawal (*n* = 20) completed a standardized scale for measuring general family features. The results revealed that the families of individuals with ESW mainly differed from the control families on the dimension of cohesion: the statistically significant lower scores reported by the parents in the ESW group indicated that there was little interaction between family members, in particular about problems and emotions (Suwa et al., 2003). In a similar vein, Nonaka et al., (2019, 2021) noted that while parents of persons with ESW essentially are equipped by a good behavioral repertoire (in terms of reinforcement and punishment strategies), they seem to be more detached from their offspring in the sense that they avoid to communicate about “the elephant in the room.”

Throughout development, internalizing psychopathology in young people has been associated with low levels of warmth—or its counterpart: high levels of rejection—and high levels of psychological control (Pinquart, 2017; Rose et al., 2018), and although evidence is currently lacking it is plausible to assume that similar parenting behaviors are relevant for young people’s persistent withdrawal tendencies. When zooming in on ESW, there are at least two specific parenting behaviors that may be of particular interest. The first one is overprotective parenting, which refers to parents who are highly vigilant, supervising, and controlling toward their children and discourage them to engage in autonomous behavior (Thomasgard & Metz, 1993). In the psychological literature, parental overprotection has been mainly linked to anxiety disorders (e.g., Van der Bruggen et al., 2008), but it is obvious how overprotection could also be involved in ESW, as this type of parenting is likely to promote dependency and hinder the young person to function separately from his/her parents. Interestingly, there is some support for the notion that overprotection is associated with social isolation and loneliness, which can be regarded as proxies of ESW (Burns et al., 2022).

A second type of parenting that bears relevance to ESW is indulgent parenting, which is referring to pampering actions that have the purpose to ease and sweeten children’s life. Motives have to do with compensation behavior for negative family circumstances (e.g., divorce, less availability because of high workload), feelings of compassion (because of all the demands placed on young people), and intentions to act as a ‘good’ parent (in comparison to one’s own parent or other parents in the community; Wolford et al., 2020). Just like overprotective parenting, indulgent parenting may hinder the development of autonomy and impede the young person’s transition to adulthood (Cui et al., 2016). So far, no study can be found demonstrating that indulgent parenting is involved in ESW. However, in the ‘hikikomori’ literature, the role of ‘*amae*’ has been repeatedly discussed as a relevant family factor (Doi, 1973). *Amae* refers to the phenomenon of young people showing helpless, desperate, selfish, and demanding behaviors with the expectation that these will be accepted and satisfied by parents and others (Behrens, 2004). ESW might be seen as a radicalized form of *amae*: young people (fully) retire from society, vegetate at home, and parasite on the resources and care provided by their parents.

All in all, it can be concluded that adverse family processes seem to play a significant role in the development and maintenance of ESW: young people who display consistent withdrawal tendencies more often have parents who suffer from some kind of psychopathology, come from families characterized by less communication and cohesion, and are subjected to detrimental rearing practices (including low warmth/rejection, overprotection, and indulgence; Funakoshi & Miyamoto, 2015). An important note should be added to qualify this conclusion. That is, most research on this topic does not allow to draw conclusions on directionality and so it remains unclear whether deleterious family factors play a causal role in the origins of ESW, whether such family variables merely reflect a parental response to young people who already display strong withdrawal tendencies, or whether both scenarios apply, meaning that the link between family factors and ESW in offspring is bidirectional in nature.

#### Bullying and Peer Victimization

Bullying and peer victimization are essentially two sides of the same coin: the former term refers to the actor, while the latter refers to the recipient, but both are part of the same process that involves interpersonal behaviors which have the aim to physically, psychologically, or socially harm another individual (Eisenberg & Aalsma, 2005). The perpetrator—the young person who engages in bullying—may show a variety of actions, such as ostracism, verbal and physical aggression, and gossiping and tale telling (‘live’ or via electronic devices), that are all damaging for the victim. It is clear that bullying and peer victimization are associated with a variety of psychopathological outcomes and these also include social adjustment problems, such as withdrawal and loneliness (Arsenault, 2018; Hawker & Boulton, 2000; Moore et al., 2017).

A good illustration of a study examining the link between peer victimization and withdrawal has been provided by Barzeva et al. (2020). In this research project, a survey was administered to assess prospective relations between social withdrawal (child self-report and parent report), peer victimization, and a number of other relevant variables (i.e., social anxiety and peer acceptance) in a mixed cohort of community and clinically referred adolescents (*N* = 2772) who were assessed three times during a five-year period at age 11, 13, and 16 years. Peer victimization appeared to play a significant role in the formation of the withdrawn behavior of these youths: that is, earlier peer victimization was associated with higher levels of withdrawal at age 13 (child and parent report) and age 16 (only parent report), whereas withdrawal at age 13 also increased the risk for peer victimization at age 16 (only parent report), suggesting that there might be a reciprocal relationship between these variables. On the basis of the results, the researchers concluded that negative peer experiences can be considered as a central factor influencing social withdrawal during adolescence (see also Matthews et al., 2022).

Unfortunately, among all the evidence, few studies can be found that focused specifically on the link between bullying/peer victimization and ESW. One notable exception is an investigation by Krieg and Dickie (2011) who compared 24 participants with hikikomori (aged 14 to 32 years) and 60 control participants with regard to a number of psychosocial variables, including attachment, parental rejection, and—most pertinent here—bullying. The results indicated that the adolescents and adults with hikikomori more often reported to have experienced bullying, in particular during their early adolescent years. In another study, Beccaria et al. (2022) administered a survey to identify potential risk and vulnerability factors of social withdrawal in an Italian sample of 13- to 20-year-old students. These researchers found that a substantial proportion of the students reported experiences of disdain (19%), insult or teasing (15%), exclusion from the group (14%), and other forms of (cyber)bullying (7%) and this indicates that these negative peer interactions were one of the reasons for why they had decided to restrict social contacts, to the point of retreating at home.

Briefly, bullying and peer victimization are likely to promote withdrawal tendencies in young people and hence also seem to represent a factor that plays a role in the development of ESW (see also Choi et al., 2022). Such negative social experiences threaten the fundamental need to belong, undermine a person’s self-esteem, and have a negative impact on the perception of the meaning of life (Stillman et al., 2009).

#### Internet and Use of Digital Media

In contemporary society, the use of devices such as smartphones, tablets, and computers and, in its wake, the employment of the internet have all become widespread. In particular for the younger generations, screens and a computerized world are an important element in life. The use of the digital devices and the internet certainly has a number of positive features that may stimulate young people’s development, but there are also a number of downsides (e.g., Bremer, 2005; Guan & Subrahmanyam, 2009), and these become clearly evident in people with ESW.

For one thing, the common availability of computer devices and the advancement of the information and communication technology via the internet allow people to stay at home and to conduct all kinds of daily activities virtually instead of physically. For example, during the COVID-19 pandemic, people in many countries faced one or multiple lockdowns, but while they were restricted to their houses in order to prevent the spread of the virus, the digital technology enabled them to continue their life by engaging in telework, telehealth, online learning, and online shopping (Mouratidis & Papagiannakis, 2021). Thus, all these ‘e-activities’ will make it possible for persons with ESW that some of the primary necessities of life can still be met, even when the social withdrawal is so persistent that one is never leaving the house anymore.

Young people may use social media channels (e.g., Facebook, Instagram, Tiktok) to satisfy their need to belong (Bozzola et al., 2022), but there is debate on whether technology-mediated social interactions have the same potential as live social interactions.

Relevant in this regard is the ‘displacement hypothesis,’ which postulates that social problems and associated negative feelings such as loneliness and depression arise when people displace real-life relationships with online ones. Evidence for this notion was provided by Kraut et al. (1998) who followed a sample of 169 persons (in 73 households) during their first two years online. The researchers not only found that greater internet use was associated with a decline in ‘live’ social participation, but more importantly the data also demonstrated that greater employment of the internet in the long run resulted in an increase of depression and loneliness. Especially when employed to deal with social inadequacies or to escape from the social world, the use of the internet seems to be problematic and is likely to have detrimental consequences (Nowland et al., 2018).

The latter often applies to individuals who display ESW. For example, in a study of Lee et al. (2013), 41 young people with persistent social withdrawal (mean age 16 years) who had been referred to a home visitation program were compared to 239 age-matched control with regard to their (excessive) internet use. The results indicated that the computer use of youth with ESW was more than two times longer than that of control youth. More than half of them (56%) displayed problematic use of the internet and a further 9% even exhibited behavior that could be qualified as an ‘addiction’ as their internet use had increased to such a frequent and intense level that it interfered significantly with the daily functioning. In the control group, these percentages were considerably lower, namely 36% and 3%. Another investigation by Tateno et al. (2019) obtained similar findings in a non-clinical sample of college and university students (*N* = 478) who completed a survey, which contained questions about internet use as well as standardized scales measuring ESW (hikikomori) symptoms, smart phone addiction, and internet addiction. It was found that participants at risk for ESW not only spent more time on the internet but also displayed higher levels of smartphone use and internet addiction as compared to participants who were not at risk for ESW. Furthermore, small to modest but statistically significantly positive correlations were noted between ESW symptom levels and both types of digital addiction, indicating that stronger social withdrawal tendencies are accompanied by more problematic usage of internet and smartphone.

It should be noted that the directionality of the link between the superfluous use of internet and digital media and ESW is far from unidirectional. On the one hand, there is the contention that excessive employment of the internet and other digital equipment (e.g., smartphone) plays a causal role as it is undermining and diminishing young people’s social behavior and as such promoting withdrawal tendencies. On the other hand, it has also been argued that the frequent employment of internet and digital technology is an effect that occurs in young people who for some reason (obviously this includes the presence of psychopathology; Carli et al., 2013) have become marginalized by their peers, at school or at work, and by society in general. Both scenarios seem valid, pointing in the direction of a reciprocal relationship and suggesting that excessive internet usage is an important maintaining factor in the withdrawal behavior of contemporary young people (Kato et al., 2020b). Meanwhile, there are also echoes in the literature suggesting that the internet and digital media are a positive element in the life of persons with ESW. For some of them, digital platforms constitute a ‘social lifeline’ through which they can actively interact and communicate with others in an easy, anonymous, and less risky way (because face-to-face contact with other people requires more skills; Wong, 2020a, 2020b).

## A Developmental Psychopathology Framework for ESW

It can be concluded that ESW is a multiplex phenomenon that appears in many disguises. Originally described as ‘hikikomori’ and considered to be a culturally bound syndrome that is deeply rooted in the habits and mannerisms of the Japanese society (Teo, 2010; Teo & Gaw, 2010) or even the Eastern culture in general (Wong et al., 2019), it has become clear that prolonged social withdrawal is present worldwide and hence should be viewed as a global phenomenon (Kato et al., 2012, 2019). In the current review, we mainly focus on the persistent social withdrawal tendencies of young people who face the developmental transformation to adulthood. In late adolescence and early adulthood, all human beings enter a process of transition during which they gradually leave the shelter and safety of their family to move to an independent life with its own roles and responsibilities (Berk, 2022). ESW seems to seriously disturb this transition and can even be seen as a sign indicating that the normal developmental process has stagnated and gone awry. As such, it is important to understand the nature of ESW and to know more about its etiology. Previous reviews (Kato et al., 2019; Li & Wong, 2015b; Nonaka et al., 2022; Teo, 2010) identified quite a number of variables that seem to be involved in persistent social withdrawal. In this review, we have tried to provide a comprehensive picture of the factors that are relevant for the origins of ESW and to highlight their contributions to the phenomenon.

In this section, we consider ESW from a developmental psychopathology perspective. Developmental psychopathology is the study of the development of psychological problems within the course of life (Cicchetti, 2006). This scientific framework is based on four core principles. The first principle assumes that there is a continuum between normal and abnormal behavior, which means that a psychopathological condition does not constitute a qualitative distinct category (a disorder or problem that is either present or absent), but rather should be seen as an aberration of a normal developmental process. As such the condition can best be regarded as extreme expressions on a dimension: it is essentially a matter of behavioral deficit or excess—the quantity of the behavior (which also includes emotion and cognition) is substantially lower or higher than what is expected on the basis of the individual’s age or developmental stage. For example, although there is a tendency in the psychological literature to view social withdrawal as a negative phenomenon (which is obviously true for cases where the tendency to isolate oneself has become too dominant or persisting), it is good to be aware that such behavior may also have positive and adaptive features, as we have indicated earlier. Seeking seclusion might be particularly useful when works need to be done, when one prefers to reflect and think, for the purpose of resting without disturbance, or for the sake of privacy. Bucholz (1997) even considers ‘alonetime’ as essential for a healthy development and argues that the ‘need to be alone’ is just as important as the ‘need to belong’ and this seems especially the case during late adolescence and early adulthood (Coplan et al., 2019b).

The second principle pertains to the fact that skills and abilities do not occur randomly but progress in roughly the same order during developmental stages. Although the timing may differ from one young person to another, during each stage the individual faces developmental hurdles that need to be taken before advancing to the next stage. The transition from adolescence to adulthood is concerned with young people’s establishment of identity and uniqueness from others; this is achieved by developing one’s own interests, values, goals, and worldviews independent from close others as well as by gaining autonomy (Zarrett & Eccles, 2006). More concretely, this implies that the young person is gradually distancing from his or her parents (both psychologically and physically) and forming new relationships with other people (i.e., friends, partner, and other companions) outside the family. He or she also engages in schooling or training with the aim to find a suitable occupation and to develop the professional skills needed in the work environment, which ultimately will make it possible to earn money and create one’s own means of subsistence (Berk, 2022). As alluded to earlier, in ESW this process has (completely) stagnated: the young person does not reach out to other people, remains emotionally and behaviorally dependent on his parents, refrains from schooling and/or work, and hence is prone to develop some twisted sense of identity (Yung et al., 2021).

The third principle is focused on factors that play a role in the dysregulation of normal behavior (to become an abnormality) and that hinder the person to successfully handle a developmental hurdle. Developmental psychopathology assumes that psychopathological conditions are seldomly the result of a single causal factor but postulates that the etiology of abnormal behavior involves multiple factors that operate in dynamic interaction and transactions. Not only factors that increase the individual’s proneness to develop a problem or disorder (i.e., risk and vulnerability factors) are of relevance, but also factors that shield the person against a problem or disorder (i.e., protective factors). The presence of multiple risk and vulnerability factors raises the probability of a disorder or problem to occur, whereas the presence of one or more protective factors decreases the probability of such a pathological course. As described in the present review, within the context of ESW, various risk and vulnerability factors have been identified, which nicely represent the various levels described in Bronfenbrenner’s ecological systems theory (Bronfenbrenner & Morris, 2006). This theory posits that a young person’s development is not only determined by person-related factors but also by a nested structure of environmental variables, including the family, school, and peer group (microsystem), the community and mass media (exosystem), and culture and society (macrosystem). So far, most research has focused on the contribution of a single factor or a single context, with little attention to how variables add to and interact with each other in the development of young people’s withdrawal behavior. Also, the topic of protective variables has been largely neglected. One exception has been provided by Markovic and Bowker (2017) who noted that the presence of a mutual friendship provided socially withdrawn youth with positive social experiences, which ultimately improved their socioemotional functioning. This suggests that friendships matter and may buffer adverse outcomes (i.e., loneliness and depression) in withdrawn youth (Rubin et al., 2010).

The fourth and final principle pertains to the fact that psychopathology tends to change throughout the course of development. One illustration of change is that the intensity and/or frequency of symptoms of a problem or disorder might increase or decrease over time. This might be the result of a transactional process (i.e., the interplay of nature [child-related factors] and nurture [environmental influences] on developmental outcomes), new developmental challenges that are encountered and need to be resolved, or temporal changes in the balance among risk, vulnerability, and protective factors. In the literature on social withdrawal, various developmental trajectories have been observed during middle childhood to early adolescence (Oh et al., 2008), from later adolescence to early adulthood (Barzeva et al., 2019), or even across the first four decades of life (Tang et al., 2017). These studies show that withdrawal symptoms in most young people are at a stable low level (i.e., reflecting the normative development), but that there also are subgroups of youth (a) showing stable high levels of withdrawal symptoms or (b) who gradually develop such symptoms over time or—in contrast—(c) who initially display clear withdrawal symptoms but gradually become more socially involved. In addition to this, a recent longitudinal study by Hihara et al. (2022) indicated that once symptoms of ESW are in the severe range, they tend to escalate over time.

**Fig. 1:**
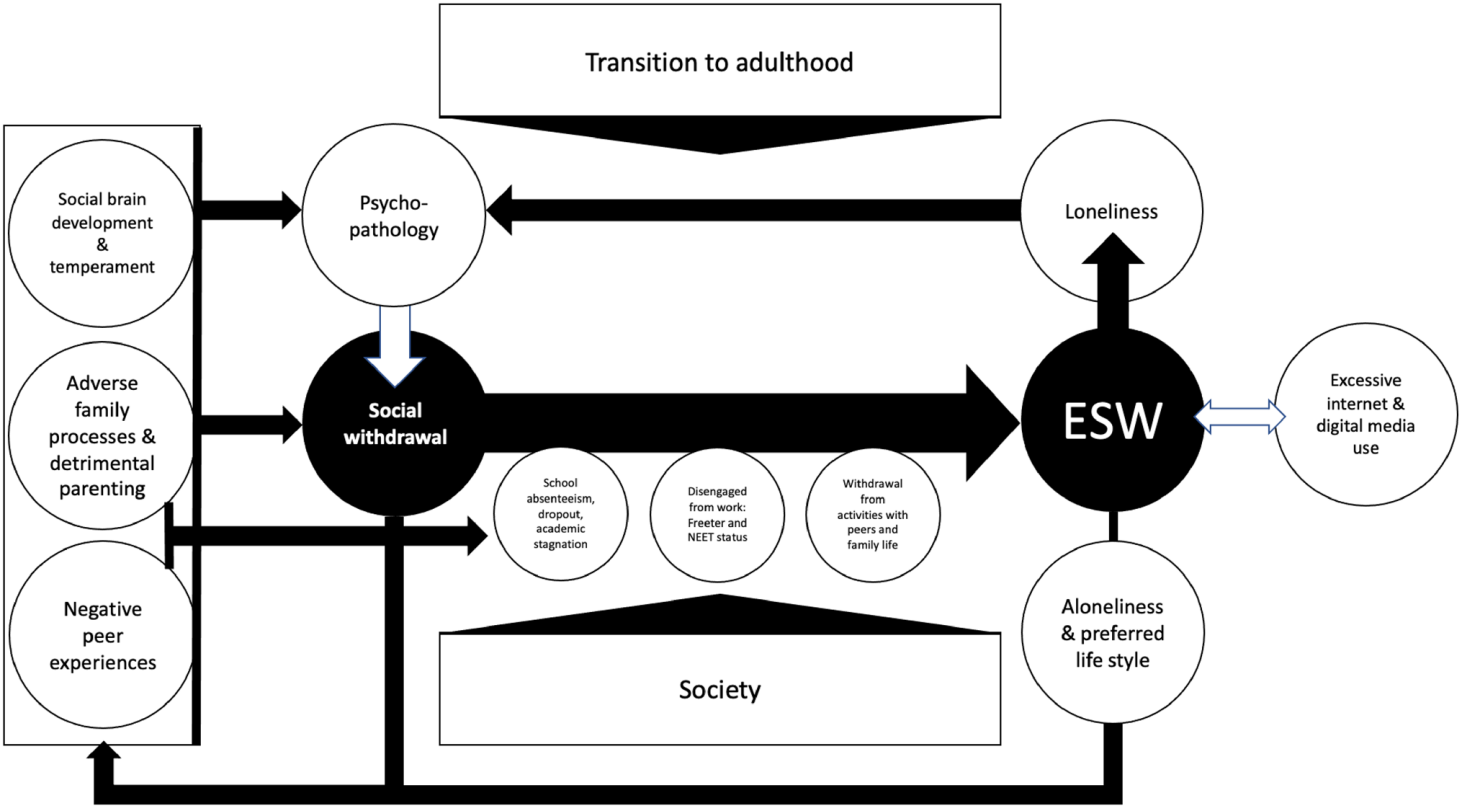
Developmental psychopathology model for the etiology of Extreme Social Withdrawal (ESW). For reasons of clarity, only main effects are shown in the model, but it should be kept in mind that developmental psychopathology assumes risk and vulnerability factors to operate in dynamic interaction and transactions (Cicchetti, 2006)

Figure 1 presents a model depicting various factors that are involved in the pathogenesis of ESW. As can be seen, person-related variables (e.g., shyness, unsociability, introversion), adverse family processes and detrimental parenting behavior, and negative peer experiences increase the probability of social withdrawal behavior to occur. Interestingly, many of these antecedent variables have also been found to be implicated in the origins of psychopathological conditions of which social withdrawal behavior is an important feature (i.e., social anxiety disorder, depression, schizophrenia, and autism spectrum disorder). In case these types of psychopathology are also present, the withdrawal behavior will be more severe and prominent, thereby increasing the likelihood for social disengagement in various domains of life: school, work, with peers, and even within the family, paving the way for ESW to develop. This scenario is even more likely to occur when young people face the transition to adulthood, a developmental stage during which the formation of identity and the acquisition of autonomy are key goals (Berk, 2022), but which may be more difficult to attain for contemporary youth because of disadvantageous societal influences (e.g., greater emphasis on schooling, difficult-to-access labor market).

As noted in our review, ESW has an intimate relation with excessive use of internet and digital media, which constitutes an important (for some young people even the only) lifeline with the external (social) world and seems to play a role in the maintenance of social avoidance behavior. Another important feedback loop that contributes to the continuation of ESW is fueled by loneliness. More precisely, the increased social isolation will threaten the need to belong and fuel feelings of loneliness, which in turn will increase negative affect and psychopathology, and in its wake perpetuate withdrawal tendencies. Even a young person who initially does not suffer much from the social isolation—as the person likes to be on his or her own and/or prefers a solitary life—is prone to get trapped in a downward spiral of ever-increasing social withdrawal and its negative correlates, because this behavior is likely to elicit and/or consolidate adverse family processes and detrimental parenting as well as negative reactions from peers.

This developmental psychopathology framework may be helpful to understand why some young people have difficulties in making the transition to adulthood and increasingly withdraw themselves from social life to adopt a hermit-like lifestyle. As we have seen, in a substantial proportion of the youths, the persistent social withdrawal behavior may have its roots in some psychiatric condition. Meanwhile, it is good to be aware that ESW is a phenomenon that is also fueled by environmental influences (e.g., detrimental parenting, negative school climate) and societal trends (e.g., increased use of internet and digital media, emphasis on schooling, changes in the labor market). This implies that when trying to resolve the problem, a child-focused psychiatric approach might be too narrow. In the next section, we will provide a summary of possible intervention approaches for ESW.

## Interventions for ESW

Given the nature of ESW, it may be quite difficult to engage young people with ESW in an intervention. Early intervention and even prevention might be most productive. The most appropriate moment for doing this seems to be at the middle school or high school levels—when education is still compulsory in most countries—and where students who are frequently absent or even drop out can be readily detected. Of course, a good functioning attendance officer needs to be present who then in cooperation with the school counselor can try to find out what is going on with the young person and why he/she is displaying these (early) signs of withdrawal behavior. In case bullying plays a significant role in the disengagement from school, an intervention should be conducted to tackle this problem (see for a recent review, Ng et al., 2022). Another possibility is that the young person is in a school level that does not match his/her intellectual abilities, which might be easily detected by taking a standardized intelligence test and eventually lead to an adjustment of the educational level. In case there are indications for the presence of (severe signs of) psychopathology and/or significant family problems, it seems wise to make a referral to a mental health specialist for further evaluation and treatment (Kearney et al., 2019). Secondary school may also provide an ideal infrastructure that can be used to evaluate the well-being of its pupils in a systematic way; this could be done by means of a universally administered survey that includes questions screening for psychopathology in general and the risk for (extreme) social withdrawal specifically. Such an approach would make an early detection of the problem possible and offers the school an opportunity for preventive action. The 25-item Hikikomori Questionnaire (Teo et al., 2018) and the Hikikomori Risk Inventory (Loscalzo et al., 2022) might be suitable tools for this purpose as they jointly give a good first impression of the severity and frequency of social withdrawal behavior and associated symptomatology.

Once young people have finished middle and secondary school, ESW will be more difficult to detect and remain largely hidden from the outside world. These young people mostly live with their parents; some may have initially started with further education but gradually disengage from higher education as they do not find their way. After aborting education, youth may adopt either a freeter or a NEET status, which probably depends on how parents deal with the issue of their young adult child not being involved in training or education and staying at home all the time without a clear mission. For parents, it may be difficult to determine whether the withdrawal behavior is representing a normative phenomenon of a young person encountering some problems to take the next step in life or should be seen as a sign of a stuck developmental trajectory. Even when the social withdrawal becomes more prominent and the young individual obviously suffers from a significant problem, parents may tend to decline from seeking help for their socially isolated youngster (Li & Wong, 2015b). Reasons for this reluctance might be the shame, embarrassment, and stigma associated with the disclosure of having a child with ESW or another mental problem, lack of knowledge about psychopathology in general and the condition of ESW in specific, and also the fact that the problem mostly causes little harm or inconvenience to other people (Suwa et al., 2003).

Psycheducation might be a first step to promote general awareness of the phenomenon in the community and help parents understand the seriousness of the problem while at the same time inform them that other young people and their families struggle with the issue. For this purpose, Teo et al. (2015c) developed a 3-h multi-component program consisting of a film screening, a lecture, and a question-and-answer session, all intending to inform the general public about ESW. The program was tested at three events that were organized in cities in the Midwestern part of the USA and that (via flyers, emails, and website announcements) attracted a total of 163 attendees. A post-event survey indicated that following the meeting, participants had clearly gained knowledge about ESW, with the majority of them acknowledging the possible problematic nature of the phenomenon and prompting intentions for utilizing mental health services. Altogether, these results are promising and we think that this type of program would also be useful to inform general practitioners about ESW as they are often the ones that refer young people and their families to mental health professionals. And, they too might be less informed.

After a referral has been made, it may still be challenging to engage the young person with ESW in an intervention or treatment (Su et al., 2021). Therefore, the threshold for the intervention should be kept as low as possible. In many countries, home visitation programs have been launched to detect, evaluate, and eventually treat socially withdrawn people, which seems to be a logical approach given the nature of the problem (e.g., Japan: Funakoshi et al., 2022; Hong Kong: Yuen et al., 2019; Korea: Lee et al., 2013; Spain: Malagón-Amor et al., 2020). Oftentimes (initially) social workers—and not clinical psychologists and psychiatrists—are involved to prevent pathologizing and hence to maximize the chance of a positive acceptation of the help (Li et al., 2018). An alternative is provided by the internet: Given the fact that many young people with ESW frequently employ this medium, it seems plausible to provide them with support, counseling, and interventions online (Chan & Lo, 2014a; Yoshikawa et al., 2021), although not much empirical work has been done to evaluate the effects of such an approach in younger individuals in general and ESW in specific. One exception is a study by Bailey et al. (2018) who developed an online social networking intervention for young people at risk for suicide of whom many were withdrawn and socially isolated. The internet platform was used to provide (a) individually-tailored interventions that had the purpose to promote young people’s coping and self-regulation skills (e.g., cognitive-behavioral therapy, mindfulness training) as well as (b) an opportunity for peer-to-peer contact which was meant to reduce their thwarted sense of belongingness and associated feelings of loneliness.

As it can take some time to establish a trustful working relationship with the withdrawn young person, one could make a start with an intervention targeting the family members. As noted earlier, the communication between parents and their child is often suboptimal: parents are often rejective and negative toward their child, tend to evade sensitive topics, and display indulgent behavior to avoid emotional outbursts of their offspring. Kubo et al. (2020, 2021) developed a special intervention program for parents of young people with ESW, with the aim to increase knowledge about the condition, offer tools for how best to react to their withdrawn offspring, and change malfunctional family interactions (using elements of cognitive-behavior therapy). Pilot investigations have indicated that the program has positive effects on parents’ interactive skills and supportive actions toward offspring with ESW, although it should be emphasized that the effect sizes for these changes were quite modest and certainly did not lead to a substantial improvement of young people’s withdrawal behavior.

To achieve the latter goal, an individualized, child-focused approach is a *condicio sine qua non*. As noted earlier, investment in establishing the contact and alliance with the withdrawn young person is extremely important as a too invasive approach is likely to result in resistance, non-acceptance of the offered help, and consolidation of the withdrawal behavior (Wong, 2009). In this regard, Yong and Kaneko (2016) suggested to begin interventions with talking sessions that allow the withdrawn young people to freely talk about themselves and their situation with the aim of facilitating self-reflection, which eventually may lead to self-motivation to change. Once open to treatment, most withdrawn people prefer some kind of individual psychotherapy (53%), while other treatment options were found to be less desirable (i.e., pharmacotherapy: 19%, combined psychotherapy/pharmacotherapy: 9%, exercise: 6%, group psychotherapy: 3%, spiritual activities: 3%; Teo et al., 2015a). The exact content and format of the therapy may be variable, but it is important to target those problems and issues that hinder the young person to develop his own identity, coping resources, and skills so that he/she will be able to (re-)connect to and participate in the social world. A first step is to eliminate prominent psychopathology (which is ‘feeding’ the withdrawal behavior) by means of evidence-based interventions. In the case of affective problems, such as social anxiety disorder and depression, cognitive-behavior therapy or interpersonal therapy (possibly in combination with pharmacotherapy, preferably selective serotonin reuptake inhibitors) seems to be clearly indicated (Klein et al., 2007; Soler & Weatherall, 2007; Stangier et al., 2011). Fluid psychotic states also often require treatment with medication (Bjornestad et al., 2020) and such an intervention could also be helpful for young people with severe autism spectrum problems (Popow et al., 2021). Social skills training could be particularly helpful if the young person has clear deficits in the ability to adequately perform social behaviors (De Mooij et al., 2020; Kratochwill & French, 1984).

Interestingly, a comprehensive review by Yung et al. (2021) identified five domains that could be a focus of intervention in (young) people with ESW: (1) Connectedness, which involves the establishment of connections with peers, receiving support from others, and becoming part of the community; (2) Hope and optimism, which pertains to belief in one’s ability to recover, finding the motivation to change, think positively about the future, and pursue goals and dreams; (3) Identity, which has to do with (re)building and (re)defining a positive sense of identity and getting rid of the stigma associated with the withdrawal; (4) Meaning in life, which concerns rebuilding one’s life, engaging oneself in worthwhile relationships, and setting and trying to achieve relevant goals, thereby promoting the quality of life; and (5) Empowerment, which encompasses taking personal responsibility and control over one’s life. It makes sense that by addressing each of these domains, the situation of a young person with ESW can be improved and help him/her on the way to recovery. So far, systematic evaluation of the efficacy of psychotherapeutic intervention for young people is largely lacking and this seems an important direction for future research.

Three specific aspects may require additional special attention when working with young people with ESW. The first aspect concerns the refusal to go to school, which is often a first sign of a young person’s withdrawal from normal life. Given that school refusal may be based on different motives, a careful assessment is necessary in order to plan the appropriate intervention(s), which may include child-, family-, and school-focused components (Elliott & Place, 2019; Kearney et al., 2019). The ultimate goal is that the young person returns to school and eventually graduates, but the social integration at school is just as important as this has also been shown to be predictive of later success in education and employment (Parker et al., in press). The second aspect that is particularly relevant for young adult individuals with ESW has to do with the restoration of work status. Ismail (2020) describes three cases of young NEETs who after some preparatory sessions engaged in a special voluntary program in which they were trained in a variety of skills that would help them to access education or the job market and that would navigate them through study or working life, thereby reaching some form of economic independence. Business and government should embrace this type of programs and create more job opportunities for young people who run the risk of dropping out the workforce in order to keep them engaged in society (P.W.C. Wong, 2020a, 2020b). The third and final aspect pertains to the excessive employment of internet and other digital media that is frequently exhibited by young people with ESW. Chang et al. (2022) noted that multi-level counseling and cognitive-behavioral therapy (in combination with pharmacotherapy) currently are effective psychological treatment options for youngsters with this type of addiction problem, although it remains to be seen whether the efficacy of these interventions is similarly effective in young people who also display ESW.

**Fig. 2:**
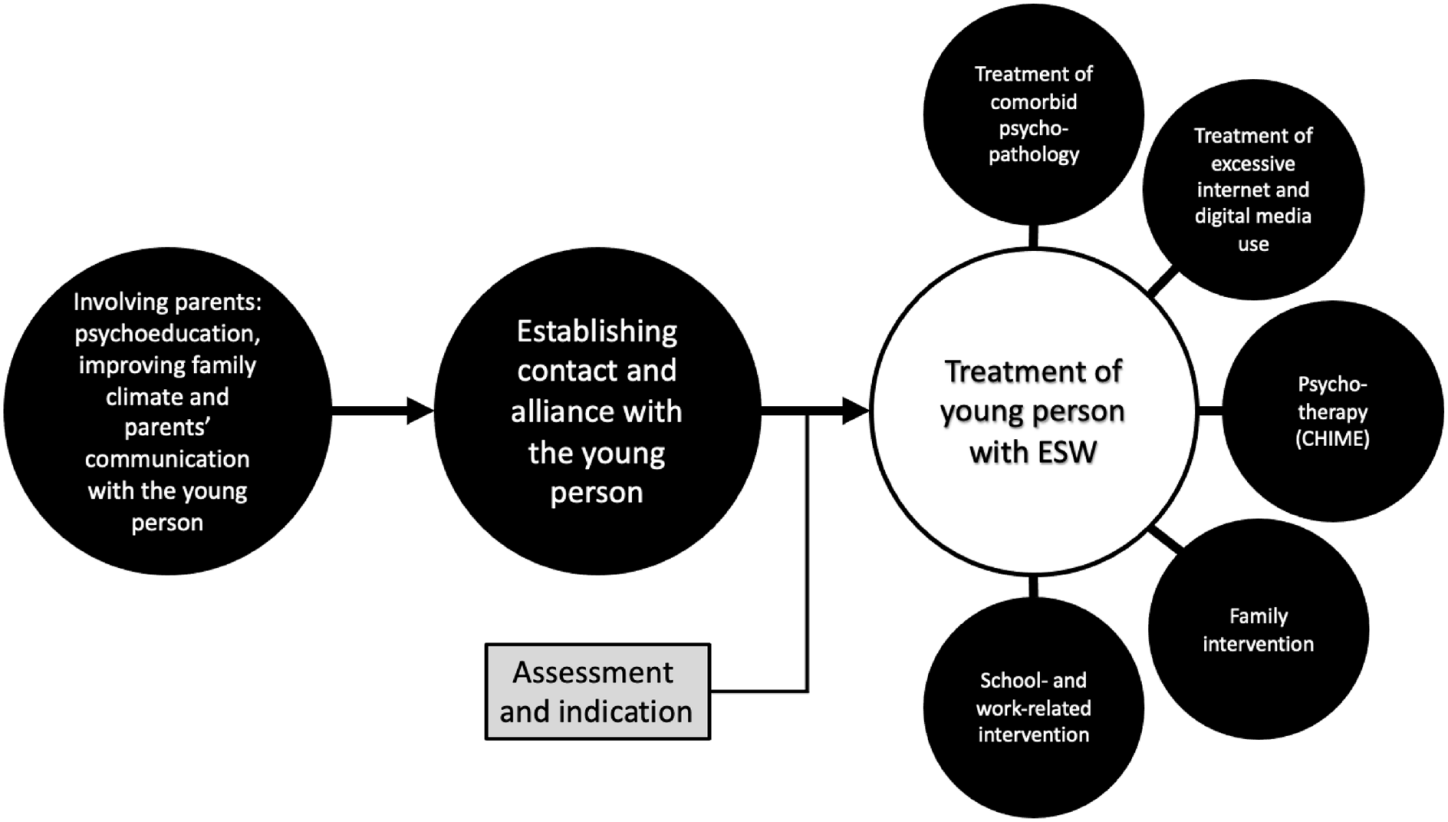
Guideline of an intervention for a young person with Extreme Social Withdrawal (ESW) and an overview of various components that need to be included in such a treatment

In sum, given the multifaceted origins of ESW, it is not surprising that interventions for this problem should consist of multiple components (see Fig. 2). A crucial step to take is to get contact and build alliance with the young withdrawn person. It may be wise in this regard to start with the parents in order to create a more favorable family climate that may also help the young person become more receptive for the upcoming intervention. Following this, the contact with the withdrawn young person needs to be established: A patient, non-invasive approach should be taken in which the youngster is invited the talk about himself and his situation with the aim of prompting self-reflection and ultimately self-motivation to change. This also gives the caregiver the opportunity to become acquainted with the young person, his situation, and in specific the factors that contribute to the social withdrawal behavior. On the basis of this assessment, indications for intervention can be made and eventually the relevant treatment components can be implemented.

## Conclusion

ESW refers to the phenomenon of young people who increasingly withdraw themselves from the external world and seclude themselves at home or even retire into their own bedroom or other isolated place. Not so long ago, we would look at ESW as some exotic, culture-bound, Japanese peculiarity, but there is increasing evidence indicating that this is a global phenomenon. In itself this is not so strange, as many of the factors underlying the problem seem to be universal, which find fertile grounds in which to thrive in the affluent parts of the world, in particular. A large national newspaper in The Netherlands recently covered the topic with a large 4-page article in which psychologists and psychiatrists signaled its presence in the Dutch mental health system (Effting, 2022). Such popular dissemination is good as they inform the general public—including parents and young people as well as clinicians—about this (clinically) relevant issue. It is also positive to see that ESW is receiving an increasing amount of research attention, but obviously more work needs to be done. It should be kept in mind that although the focus of the present paper was on young people with ESW, a great amount of the research summarized has been conducted in older participants. Thus, important directions for future studies would be to further explore etiological factors in (larger) populations of adolescents and young adults, to develop effective ways of screening and measuring the problem in this age group, and most importantly to evaluate the efficacy and effectiveness of interventions that aim to contribute to the social and societal rehabilitation of these young contemporary hermits.
